# Supplementary material for: Molecular Dynamics Simulations Reveal Canonical Conformations in Different pMHC/TCR Interactions
Source: Cells. 2020 Apr 10;9(4):942. doi: 10.3390/cells9040942 (PMC7226950; doi:10.3390/cells9040942)
Supplement: Supplementary file 1 [file cells-09-00942-s001.zip › supplementary final/cells-742528supp.pdf]

Supporting Information

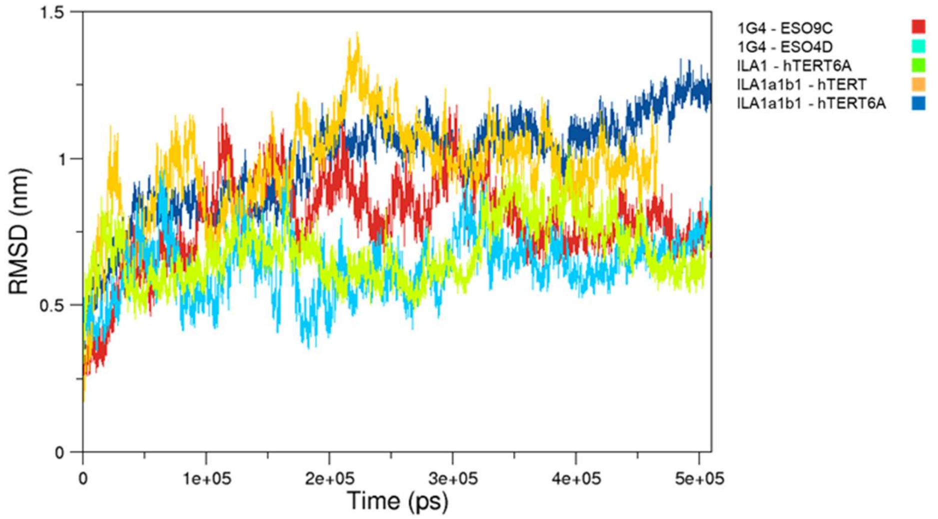

Figure S1. The RMSD computed on the alpha carbon of the systems. The RMSDs of the pMHC-TCR complexes reach a plateau after several ns. The first 50 ns were removed from the analysis.

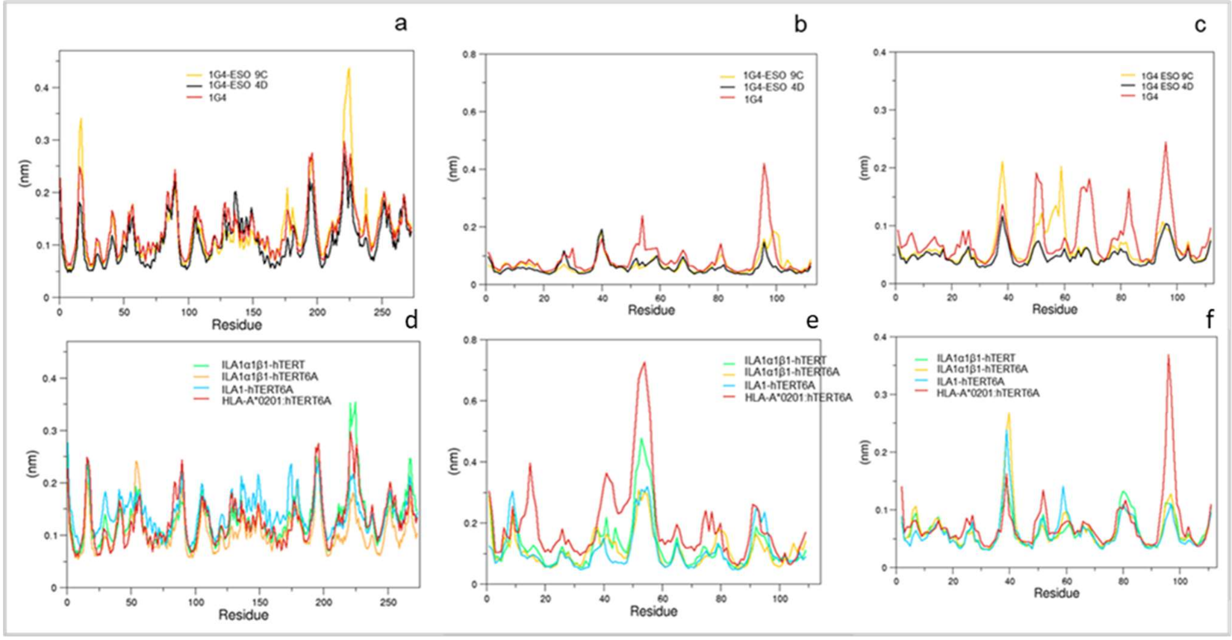

Figure S2. The RMSF of the single regions. On top the RMSF of the 1G4 TCRs, on bottom the RMSFs refer to ILA1 types. In panel a-d the HLA-A*02 RMSF is shown. In panel b-e and c-f the RMSF of the Vα and Vβ, respectively.

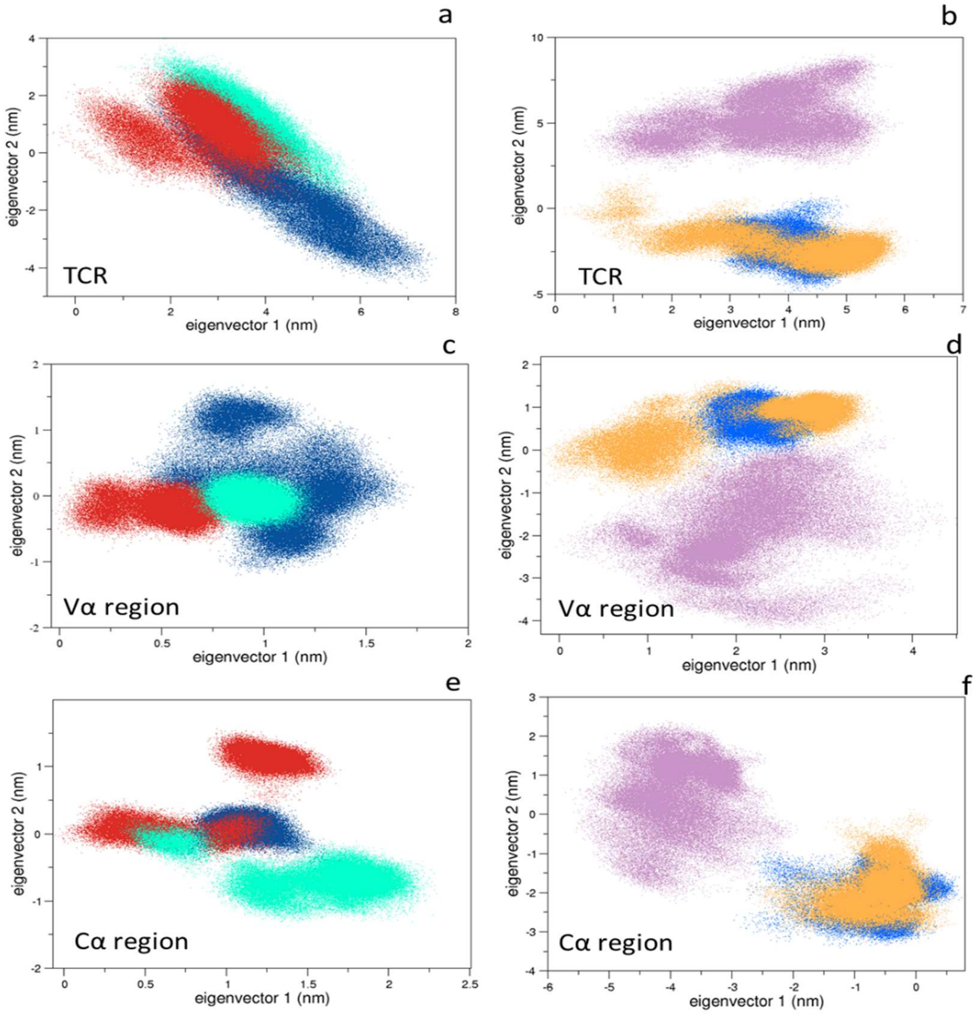

Figure S3. 2D projections of the alpha carbon of the TCR and the TCR alpha-chain. On the left the 1G4 bound-unbound states; on the right the ILA1α1β1 states. In red 1G4-ESO9C, in cyan and in blue 1G4-ESO4D and the unbound 1G4, respectively. In orange ILA1α1β1-hTERT, in blue ILA1α1β1-hTERT-6A, in mauve the ILA1α1β1 unbound.

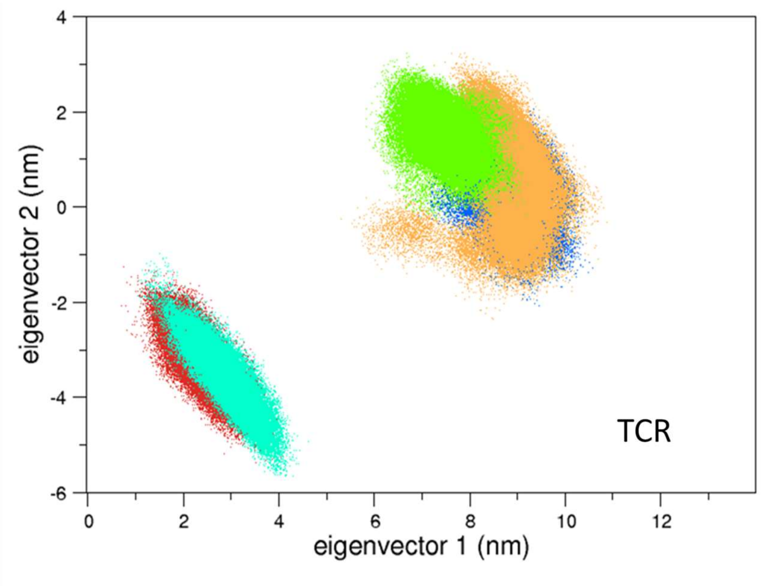

Figure S4. 2D projections of the total TCR alpha carbons of the bound states. In red 1G4-ESO9C, in cyan and in blue 1G4-ESO4D and the unbound 1G4, respectively. In orange ILA1α1β1-hTERT, in blue ILA1α1β1-hTERT-6A, in mauve the ILA1α1β1 unbound.

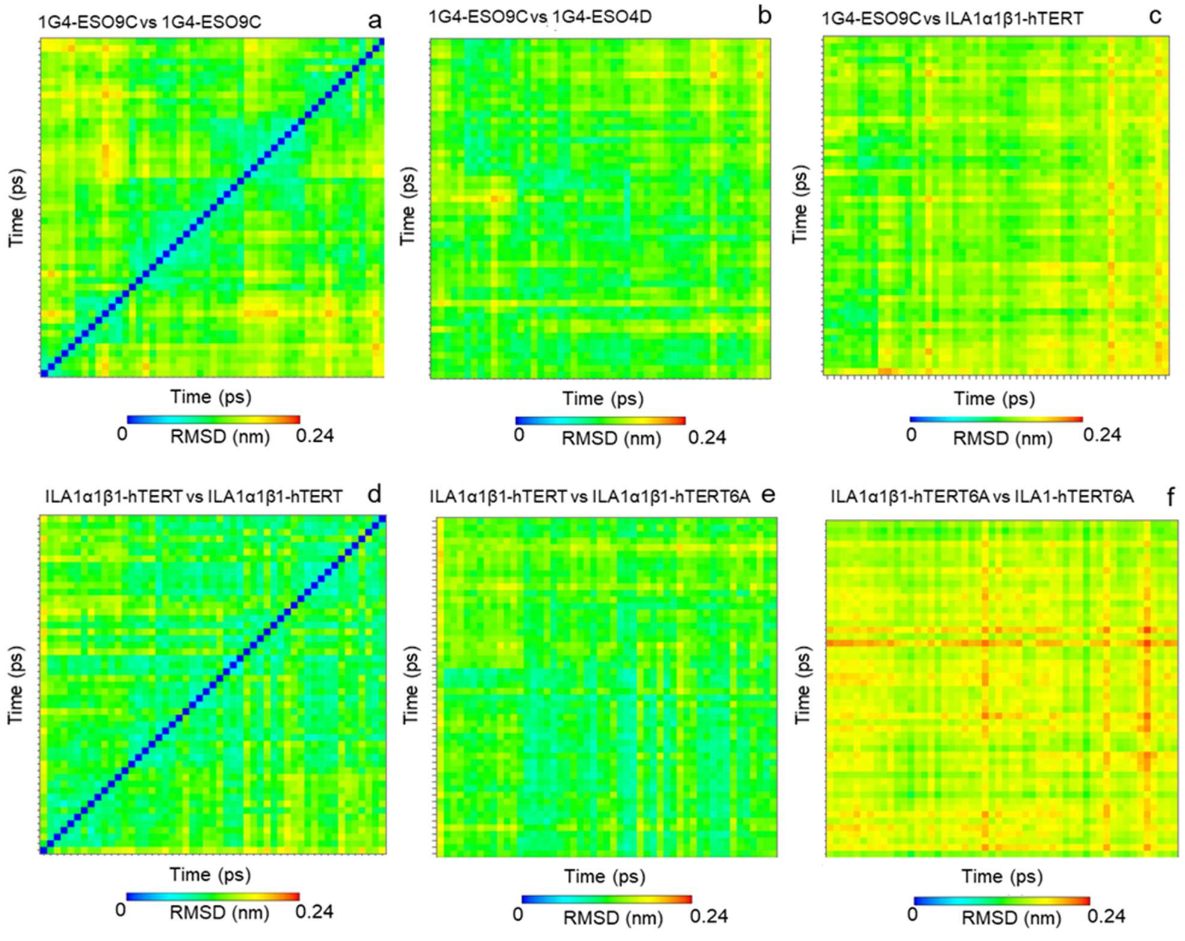

Figure S5. RMSD matrices. Each matrix represents the RMSD of the HLA-A*02:01 binding groove for each frame sampled by a refence simulation (e.i. HLA-A*02:ESO9C- 1G4, panel b) compared to another simulation (e.i. HLA-A*02:ESO9D- 1G4, panel b). In panel a and d the RMDS matrices of the reference simulation against itself are shown.

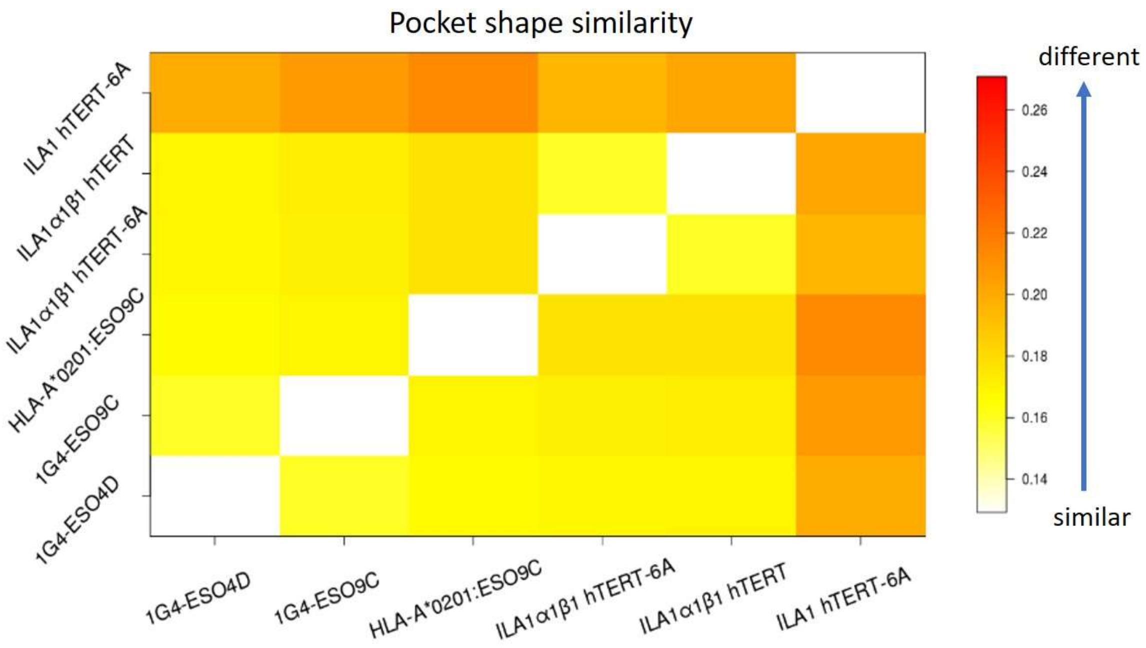

Figure S6. The Zernike Shape similarity of the binding groove. The matrix represents the mean distances, in terms of geometrical conformation, between the binding grooves. From white to red the increase distance is indicated, respectively.

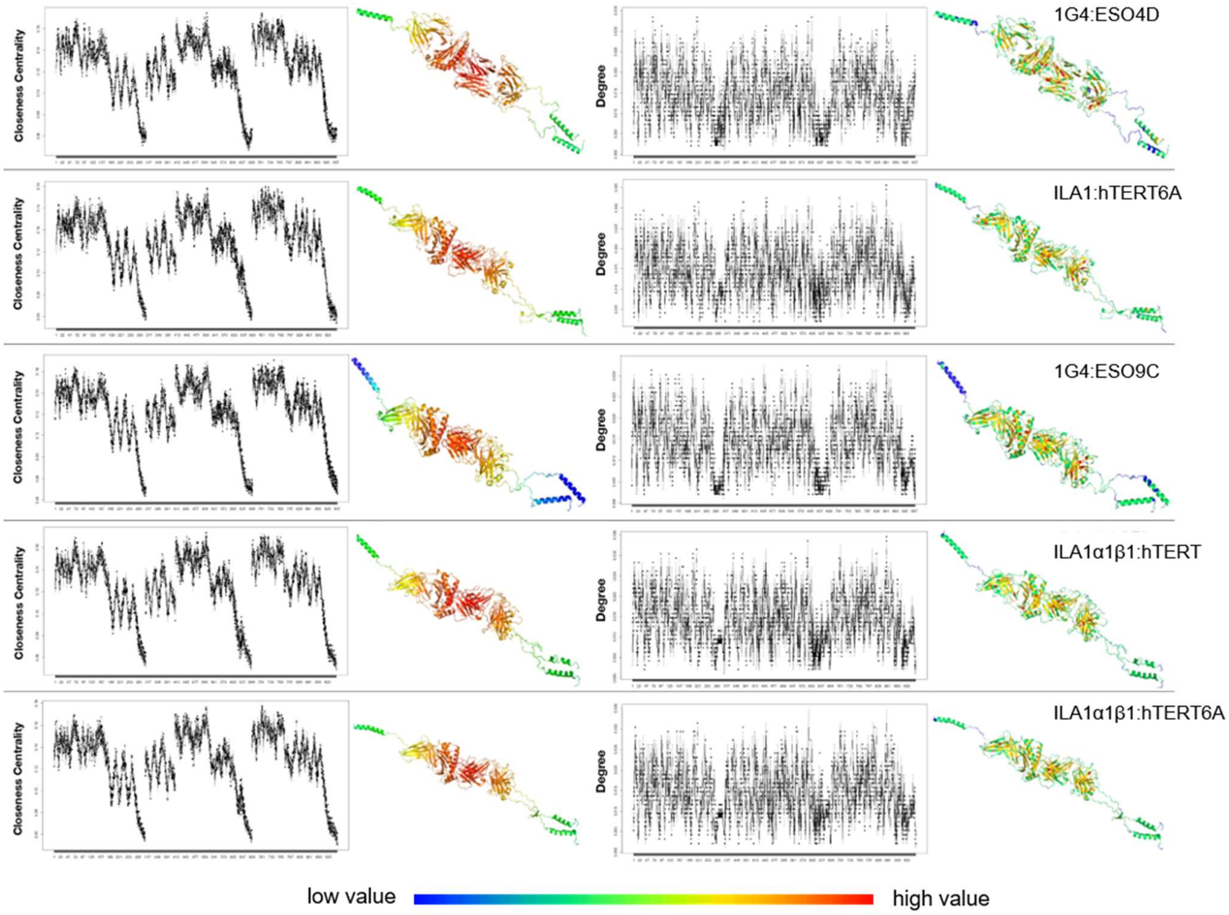

Figure S7. Network analysis. On the left, the Closeness Centrality analysis: in red the central residues mainly involved in the network connection. On the right, the Degree parameters: in red the central residues most connected. The x-axes report the residue number.

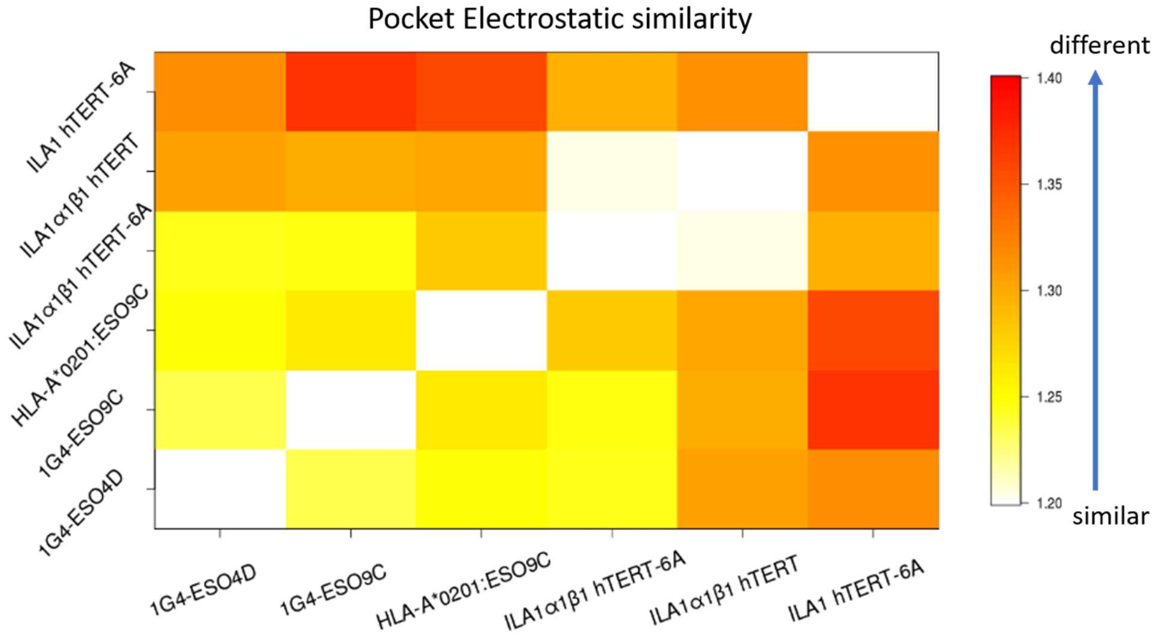

Figure S8. The Zernike pocket electrostatic similarity. The matrix represents the mean distances of the electrostatic properties between the binding grooves. From white to red the increase distance is indicated, respectively.

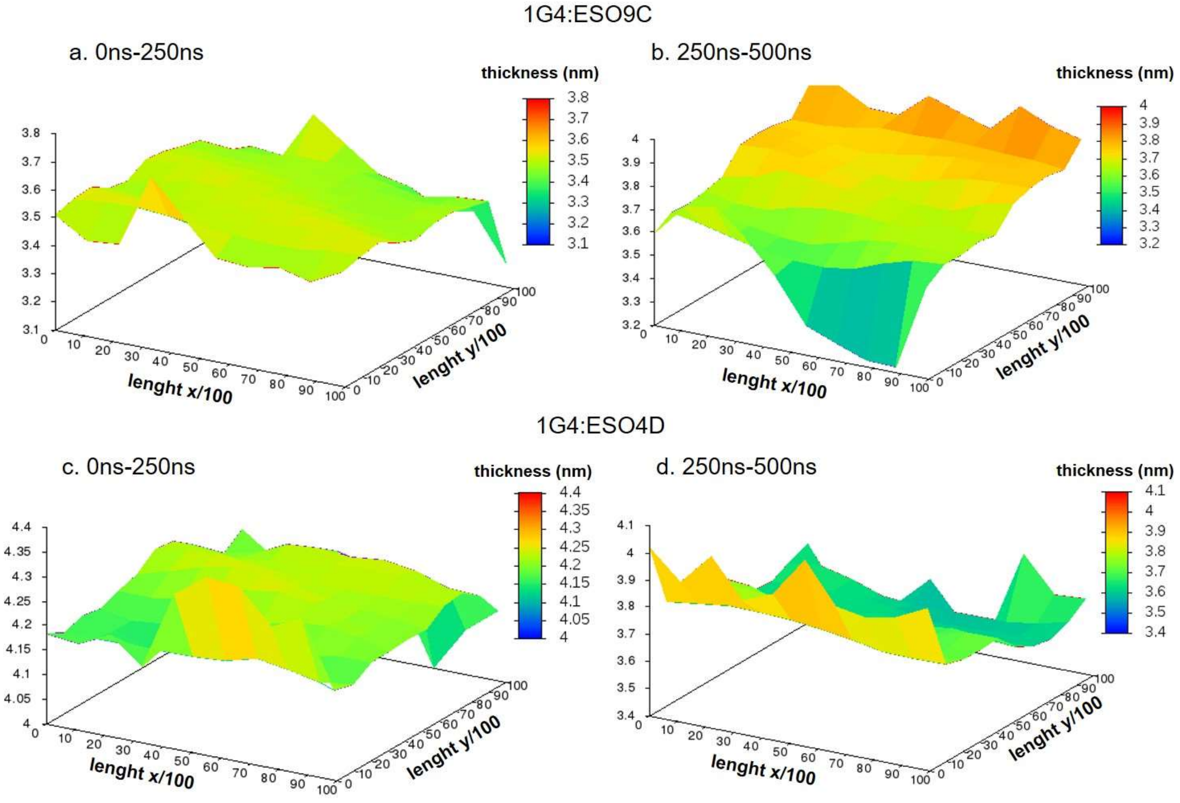

Figure S9. Membrane Thickness for the 1G4 complexes. On top, the thickness bilayer of 1G4:ESO9C; on bottom, the thickness bilayer of 1G4:ESO4D. On the left (panels a, c), the surface describing the first 250ns of each complex is reported; on the right (panels b, d) the surface for the last 250ns.

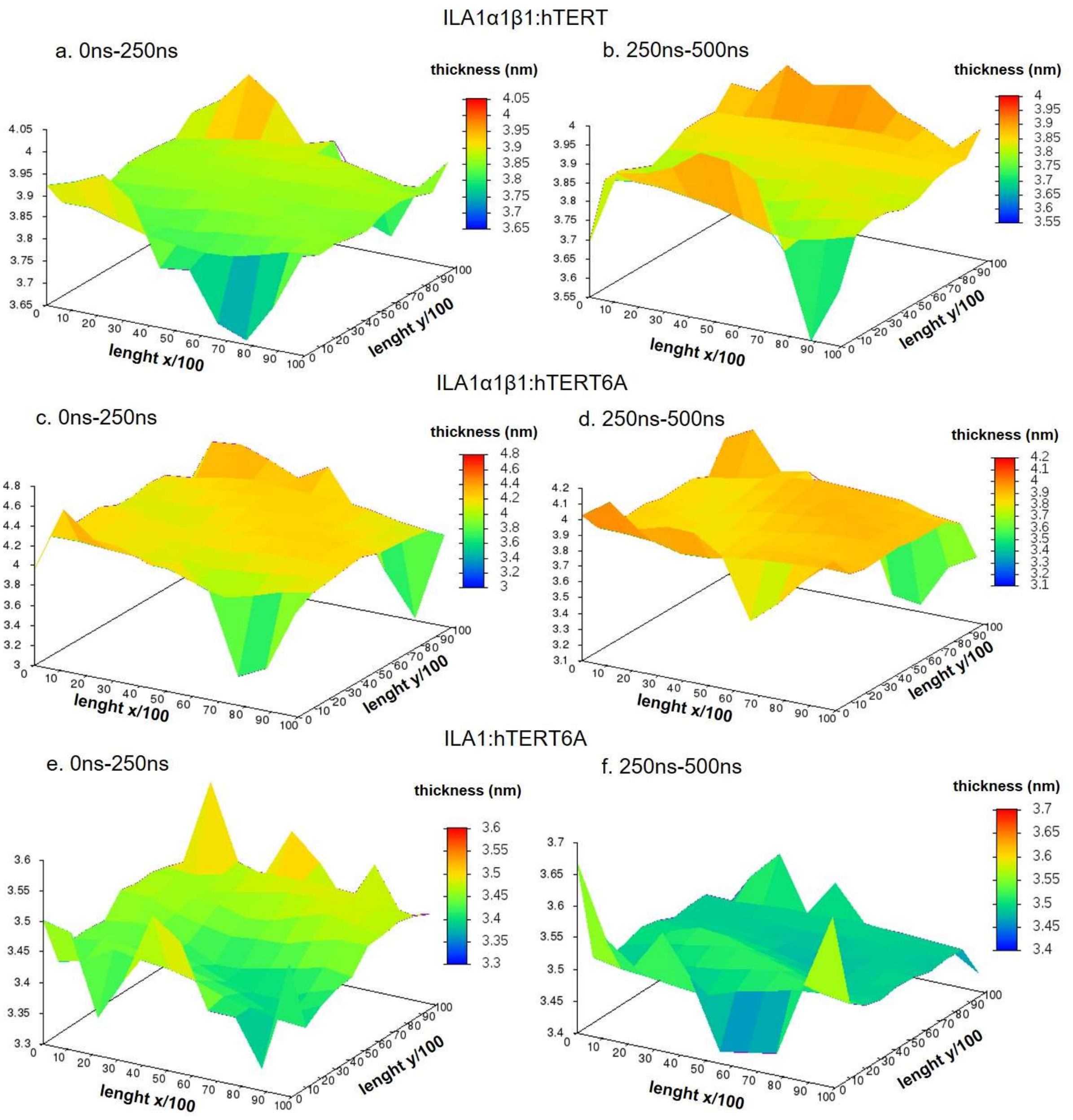

Figure S10. Membrane Thickness for the ILA1/ILA1α1β1 complexes. On top, the thickness bilayer of ILA1α1β1:hTERT, in the centre ILA1α1β1:hTERT6A , on bottom ILA1:hTERT6A. On the right (panels a, c, e) the surface describing the first 250ns of each complex is reported. On the right (panels b, d, f) the surface for the last 250ns.

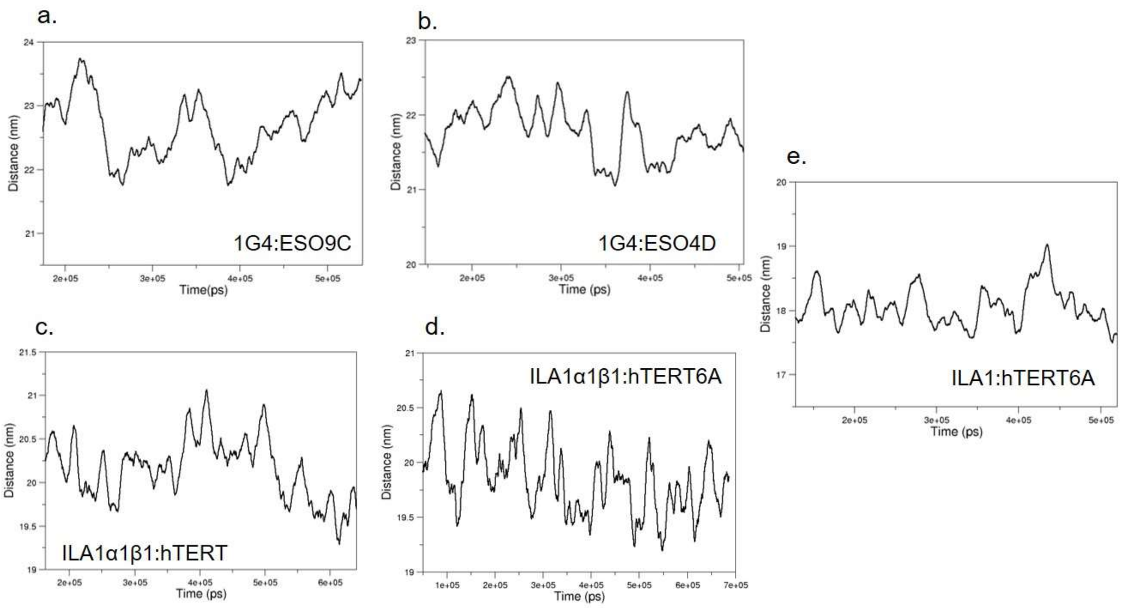

Figure S11. Distance between the bilayers. The distance between the bilayer was computed considering the headgroup of each lipid.

Table S1. The Hydrogen bonds between the peptide and the TCR are shown. The residue of the peptide which makes the h-bonds is labelled with 'P', and the associate number denotes its position in the peptide sequence.

**1G4 CDR3α**	**peptide ESO9C**	**peptide ESO4D**	**ILA1 CDR3α**	**peptide hTERT-A6**	**ILAα1β1 CDR3α**	**peptide hTERT**	**peptide hTERT-A6**
Thr 94	P5	P5	Ala 91	P2	Asp 89	P4	P4
Ser 95		P4			Ser 90	P4	P4
Tyr 99		P4			Ala 91	P3	P3
					Ala 93	P4	
					Pro 95	P4	
					Tyr 96	P4	P4
**1G4 CDR3 β**			**ILA1 CDR3β**		**ILAα1β1 CDR3β**	NO h-bonds	NO h-bonds
Val 94	P5	P5	Asp 55	P4			
Gly 95	P7		Gln 56	P4			
Asn 96	P8	P6-P8					

Table S2. Peptide Solvent exposure. The exposure for single a.a residue of the peptide is reported. On the right the average exposure for peptide. The statistic error is within ± 0.1 and 0.2.

**Peptides**	**Solvent exposure per residue (nm²)**									**TOT solvent exposure (nm²)**
	**P1**	**P2**	**P3**	**P4**	**P5**	**P6**	**P7**	**P8**	**P9**	
**1G4 ESO 9C**	1.9	1.7	1.4	1.5	2	1.5	1.2	1.7	2	15
**1G4 ESO 4D**	1.9	1.7	1.3	1.2	2.3	1.5	1.2	1.7	2.1	15±0.4
**ILAa1b1 WT**	2.4	1.8	0.6	2.1	1.5	1.3	1.4	2.2	2.5	16

**ILAa1b1 PEP5**	2.4	1.7	0.6	2	1.7	0.8	1.4	2.5	2.5	16±0.4
**ILA PEP5**	2.5	1.8	0.8	2.1	1.6	0.9	1.2	2.2	2.4	16±0.5
**ESO 9C in HLA-unbound**	1.9	1.7	1.4	1.8	1.9	1.4	0.9	1.7	2	14.8±0.4
